# Fast track multi-discipline treatment (FTMDT trial) versus conventional treatment in colorectal cancer--the design of a prospective randomized controlled study

**DOI:** 10.1186/1471-2407-11-494

**Published:** 2011-11-24

**Authors:** Jiao-Jiao Zhou, Jun Li, Xiao-Jiang Ying, Yong-Mao Song, Rong Chen, Gang Chen, Min Yan, Ke-Feng Ding

**Affiliations:** 1 Department of Surgical Oncology, Second Affiliated Hospital, and The Key Laboratory of Cancer Prevention and Intervention, China National Ministry of Education, Zhejiang University College of Medicine, 88 Jie-Fang Rd, Hangzhou, Zhejiang 310009, China; 2 Department of Anorectum, People's Hospital of Shaoxing, 568 Zhong-Xing North Rd, Shaoxing, Zhejiang 312000, China; 3 Department of Anus and Large Intestine, Second Affiliated Hospital, Wenzhou Medicine College, 109 Xue-Yuan West Rd, Wenzhou, Zhejiang 325027, China; 4 Department of Anesthesiology, Second Affiliated Hospital, Zhejiang University College of Medicine, 88 Jie-Fang Rd, Hangzhou, Zhejiang 310009, China

**Keywords:** Colorectal surgery, Rehabilitation, Colorectal neoplasms, Hospitalization, Randomized controlled trial

## Abstract

**Background:**

Laparoscopy-assisted surgery, fast-track perioperative treatment are both increasingly used in colorectal cancer treatment, for their short-time benefits of enhanced recovery and short hospital stays. However, the benefits of the integration of the Laparoscopy-assisted surgery, fast-track perioperative treatment, and even with the Xelox chemotherapy, are still unknown. In this study, the three treatments integration is defined as "Fast Track Multi-Discipline Treatment Model" for colorectal cancer and this model extends the benefits to the whole treatment process of colorectal cancer. The main purpose of the study is to explore the feasibility of "Fast Track Multi-Discipline Treatment" model in treatment of colorectal cancer.

**Methods:**

The trial is a prospective randomized controlled study with 2 × 2 balanced factorial design. Patients eligible for the study will be randomized to 4 groups: (I) Laparoscopic surgery with fast track perioperative treatment and Xelox chemotherapy; (II) Open surgery with fast track perioperative treatment and Xelox chemotherapy; (III) Laparoscopic surgery with conventional perioperative treatment and mFolfox6 chemotherapy; (IV) Open surgery with conventional perioperative treatment and mFolfox6 chemotherapy. The primary endpoint of this study is the hospital stays. The secondary endpoints are the quality of life, chemotherapy related adverse events, surgical complications and hospitalization costs. Totally, 340 patients will be enrolled with 85 patients in each group.

**Conclusions:**

The study initiates a new treatment model "Fast Track Multi-Discipline Treatment" for colorectal cancer, and will provide feasibility evidence on the new model "Fast Track Multi-Discipline Treatment" for patients with colorectal cancer.

**Trial registration:**

ClinicalTrials.gov: NCT01080547

## Background

Achieving a better quality of life for patients through enhanced recovery and shorter hospital stays in colorectal cancer treatment is becoming increasingly important.

Compared with open surgery, laparoscopy-assisted surgery has been proved by consistent evidence as a safe and effective method of colorectal cancer treatment. It also has demonstrated the benefits in reduction of surgical injury and the improvement of short-term recovery outcome [1-4].

Fast-track perioperative treatment, which is also referred as Enhanced Recovery after Surgery (ERAS), attracted much attention since Henrik Kehlet initially raised this concept [5]. This evidence-based fast-track perioperative treatment has been used for nearly a decade. The principle of the Fast-track treatment is to gain faster and enhanced recovery after surgery, by reducing unnecessary interventions which were formally involved in perioperative treatment, like reducing the fasting periods and bed rest time. Since the first time Henrik Kehlet prompted the notion, many studies have confirmed the advantages of the Fast-track treatment, especially the advantages of accelerating the short-term recovery, reducing the postoperative morbidity, shortening the hospital stays and improving the life quality [6-14].

Nevertheless, most recent researches into laparoscopy and Fast-track treatment of colorectal cancer focus on the perioperative care, which are much more related to short-term benefits, last only 1-2 weeks perioperative. In contrast, adjuvant chemotherapy takes up around 6 months of the whole colorectal cancer treatment, but has been neglected in researches mentioned above at present. So, the thoughts of combining the laparoscopy, Fast-track perioperative treatment and adjuvant chemotherapy naturally rise up, in the attempt to gain the maximum benefits covering both short-term and long-term.

Xelox and mFolfox6 chemotherapy are currently used as two comparable effective adjuvant chemotherapies for colorectal cancer, and Xelox chemotherapy is already being widely used in colorectal cancer, as it has better manageable tolerability and similar Overall Survival(OS) [15-17] versus mFolfox6 chemotherapy.

Until now, there has been no published study researching the effects of the combination of laparoscopy, Fast-track treatment and Xelox chemotherapy, which has been focused on in this FTMDT (Fast Track Multi-Discipline Treatment) trial. And we propose the new model "Fast Track Multi-Discipline Treatment" for this combination. In view of the improved rehabilitation the three component parts bring to the colorectal cancer treatment, we aim to find a more economical and effective mode for colorectal cancer treatment through this prospective randomized controlled FTMDT-trial study.

Since it has not been established whether FTMDT is superior to conventional treatment (open or laparoscopic surgery with conventional perioperative treatment and mFolfox6 chemotherapy) in colorectal cancer treatment, the following is the present study protocol.

## Methods/Design

### Study objectives

The general objective of this FTMDT trial is to assess, synthesize and discuss the feasibility of "Fast Track Multi-Discipline Treatment" model in treatment of colorectal cancer. It can be divided into 2 questions: 1. Is it safe and effective to combine Xelox chemotherapy with laparoscopy and fast-track perioperative treatment? 2. Is laparoscopy an essential component of FTMDT and how important a role does it play?

### Study design

This FTMDT-trial is an open randomized prospective and controlled study with 2 × 2 balanced factorial design. Patients eligible for the criteria will be randomized into four study groups (1:1:1:1) according to the randomized figures generated by the SPSS 16.0, after the patients signing the informed consents: (I) Laparoscopic surgery with fast track perioperative treatment and Xelox chemotherapy; (II) Open surgery with fast track perioperative treatment and Xelox chemotherapy; (III) Laparoscopic surgery with conventional perioperative treatment and mFolfox6 chemotherapy; (IV) Open surgery with conventional perioperative treatment and mFolfox6 chemotherapy.

Chinese medical insurance system ensures that, hospitalization is suitable for payment and also for patients' interest. All patients in this study will receive hospitalized chemotherapy in each cycle.

This trial has the independent "third-party" for developing the randomization.

### Eligibility

Inclusion Criteria: patients who are 18 years and older, with pathologically confirmed colon and upper rectal cancer and signed informed consent prior to enrollment.

Exclusion Criteria: Tumors which can be resected by endoscopic mucosal resection (EMR) and endoscopic submucosal dissection (ESD), history of malignancy, bowel obstruction or intestinal perforation, evidence of metastasis through physical examination, chest roentgenogram and computed tomography of liver and pelvis, acute disease and acute attack of chronic disease, psychiatric history, spinal deformity, American Society of Anesthesiologists (ASA) score ≥ IV, mid-low rectal cancer, and pregnant woman.

Adjuvant chemotherapy will be needed for III stage or high-risk II stage pathologically established colorectal cancer. The performance status of the patients after surgery will be evaluated with the Zubrod-ECOG-WHO score, before the chemotherapy start. Patients with Zubrod-ECOG-WHO score sustain ≥ 2 within 3 months after surgery will be excluded and not have the chemotherapy.

### Discontinuance criterion

The discontinuance criterion of this clinical trial as followings: 1) the trial appears causing unexpected harm or severe adverse events to participants, or the evidence that the risks outweigh the benefits, with the discontinuance decision of the ethics committees. 2) the enrollment indicates the trial can't be finished in the period of 4 years. 3) chemotherapy will be suspended for the patients with the chemotherapy adverse events more than grade 3 according to NCI-CTC AE 3.0 (National Cancer Institute Common Terminology, Criteria for Adverse Events 3.0). And the dose will be adjusted to 75% after adverse events reduce to grade 2 or lower. The chemotherapy will be withdrawn once the severe adverse events appear again.

### Primary and secondary endpoint measures

The primary outcome measure of this FTMDT-trial is the hospital stays, which is the overall hospitalization stay during treatment including both the hospital stay for the surgery and adjuvant chemotherapy.

We define the postoperative discharge day for the patients in the trial when they meet all of the discharge criteria, 1. Good pain control: NSR (Numeric Rating Scale) ≤ 3. 2. Tolerance of solid food, no need of intravenous fluid infusion. 3. Independent activities of daily living (ADL) to preoperative care level at least [18,19].

Secondary outcome measures are as follows, 1. Quality of life according to EORTC (European Organization for Research and Treatment) QLQ-C30 and QLQ-CR38 questionnaires, with the checkpoint frame, preoperation, 1 week post operation, 3-months post surgery and 6-months post surgery. 2. Chemotherapy related adverse events according to NCI CTCAE (Common Terminology Criteria for Adverse Events) Version3.0. 3. Surgical complications mainly include injury of the ureters, intraoperative transfusion, infection of incision, anastomotic leakage and readmission. 4. Hospitalization costs are calculated from the first day in hospital to the last day that adjuvant chemotherapy is finished.

### Participating centers

The FTMDT-trial is a multi-center study located in China, with 3 participating centers as follows, Hangzhou center (Department of Surgical Oncology, Second Affiliated Hospital, Zhejiang University College of Medicine), Shaoxing center (Department of Anorectum, People's Hospital of Shaoxing), Wenzhou center (Department of Anus and Large Intestine, Second Affiliated Hospital, Wenzhou Medicine College).

### Ethics

The independent medical ethics committees of the participating hospitals have approved this FTMDT-trial protocol, with the approval number: 2010LSY No.6. All the procedures of this study are under the oversight of the Chinese Ministry of Health.

### Study outline

#### Interventions

##### Surgery

Much experience has been accumulated since the first laparoscopic colectomy in 2003 in Hangzhou center, as the number of all colorectal cancer surgery has been approximately up to 350 per year, including about 30% open surgery and 60% laparoscopic surgery. Shaoxing center has done approximately 400 laparoscopic colorectal surgeries since developed in 2004 and Wenzhou center approximately 300 since 2005.

In this study, open and laparoscopic surgery will be performed according to the principles of NCCN Clinical Practice Guidelines in Oncology ™ Colon Cancer V.2.2009 and there are 8 colorectal surgeon specialists, all of whom are Attending Surgeons (Ke-Feng Ding, Xiao-Jiang Ying, Rong Chen, Yong-Mao Song, Jian-Wei Wang, Li-Feng Sun, Yong-Chuan Deng, Yi Shen). The former five of them are for laparoscopy and they have performed laparoscopic operations in colorectal cancer with a minimum of 20 as suggested by the ASCRS [20,21].

##### Perioperative treatment

Patients in groups III and IV will receive conventional perioperative treatment, while patients in groups I and II will receive the fast track perioperative treatment, which will be provided by trained doctors and nurses separately.

The essence of the fast track perioperative treatment can be represented from 3 periods, 1. Preoperation, psychological optimism and information of the fast track treatment to patients, less time fasting, oral carbohydrate loaded liquids. 2. Intraoperation, combined anesthesia consisting of epidural and general anesthesia with the use of morphine minimized as far as possible, minimal use and early removal of nasogastric tube, drains and catheter, body warming as well as intravenous fluid warming. 3. Post operation: pain management without opioid, early feeding rehabilitation, restriction of intravenous fluid infusion, early ambulation.

The comparison of fast track and conventional perioperative operation treatments are summarized in Table 1.

**Table 1:** Checklist of fast track and conventional perioperative operation treatments

Time	Fast track treatment	Conventional treatment
Preadmission	-Psychological optimism	-No psychological optimism

(After randomization)	-Pre-assessment for risk adjustment	-Pre-assessment for risk adjustment
	
	-Anesthesiologic information of combined anesthesia consisting of thoracic epidural and general anesthesia	-No Anesthesiologic information of general anesthesia
	
	-Information of the fast track treatment and the informed consent	-Information of the conventional treatment and the informed consent
	
	-Guided tour of fast track wards	-No tour
	
	-Operation schedule	-Operation schedule

Preoperation	-Bowel preparation: semiliquid diet 1 days before operation	-Bowel preparation: liquid diet 1-2 days before operation
	
	- Enemas:	-Enemas:
	
	Polyethylene Glycol-Electrolyte Powder ^® ^(Hengkang Zhengqing™, Jiangxi Hygecon Pharmacy CO., Ltd, Shangrao, CN) the afternoon before surgery,2 boxes mixing with 2,000 ml warm drinking water	Polyethylene Glycol-Electrolyte Powder ^® ^the afternoon before surgery, 2 boxes mixing with 2000 ml warm drinking water
	
	-Fasting: last meal 2 h before operation	-Fasting: last meal 10 h before operation
	
	-Complete Enteral Nutritional Emulsion Supportan (TPF-T) ^® ^(Supportan™, Sino-Swed Pharmaceutical CO. Ltd, Wuxi, CN) 600 ml or Fresubin Diabetes (TPF-D) ^® ^(Fresubin Diabetes™, Sino-Swed Pharmaceutical CO. Ltd, Wuxi, CN) 500 ml (especially for patients with diabetes mellitus) p.o. 8 h before operation	- No oral intake in the operation day
	
	- 10% Glucose 400 ml p.o. 2-3 h before operation	- No oral intake in the operation day
	
	- Nasogastric tube 0.5 h before operation for Gastrointestinal decompression	- Nasogastric tube 0.5 h before operation for Gastrointestinal decompression

Intraoperation		

-Anesthetic managemen	- Placement of epidural catheter (T6-L1), depending on the surgical resection); test-dose (3 ml of 2% lidocaine (Hefeng™, Harvest Pharmaceutical CO. Ltd, Shanghai, CN)) followed by continuous infusion (10 ml of 0.5% or 0.75% ropivacaine(Naropin™, APP Pharmaceuticals, LLC., Schaumburg, IL) according to the age and size of the patient before surgical incision	- No thoracic epidural anesthesia
	
	- Balanced Combination with general anesthesia: intravenous midazolam (Liyuexi™, Nhwa Pharmaceutical Co., Ltd., Xuzhou, CN) (0.1 mg/kg), target-controlled infusion (TCI) of propofol (Diprivan™, AstraZeneca Pharmaceutical Co., Ltd., Shanghai, CN) (4-8 μg/ml), sufentanil (Fukang™, Humanwell Pharmaceutical Co., Ltd., Yichang, CN) (0.5-1 µg/kg), rocuronium (Esmeron™, Organon Teknika B.V., Oss, NL) (0.6-0.9 mg/kg).	- Normal General anesthesia: intravenous midazolam (0.1 mg/kg), target-controlled infusion (TCI) of propofol (4-8 μg/ml), sufentanil 0.5-1 µg/kg, rocuronium (0.6-0.9 mg/kg).
	
	The patients were ventilated mechanically.	The patients were ventilated mechanically.
	
	Anesthesia was maintained propofol TCI (2-4 μg/ml), remifentanil (0.02-0.03 μg/kg/min) and intermittent boluses of rocuronium.	Anesthesia was maintained propofol TCI (2-4 μg/ml), remifentanil (Ruijie™, Humanwell Pharmaceutical Co., Ltd., Yichang, CN) (0.02-0.03 μg/kg/min) and intermittent boluses of rocuronium.
	
		As equally depth of anesthesia is also needed in conventional treatment group with no thoracic epidural anesthesia, more drug dosage of general anesthesia is used.
	
	- Morphia as little as possible	- No restriction of Morphia use
	
	- Monitoring: (Datex Ohmeda™ S/5 Anesthesia Monitor (Datex-Ohmeda Division, Instrumentarium Corp., Helsinki, Finland)) consists of electrocardiogram (ECG), heart rate (HR), respiratory rate, arterial pressure (BP), SpO2, end-tidal CO2 (etCO2), and bispectral index (BIS). - The target concentration of propofol:keep BIS between 40 and 60 to maintain adequate hypnosis. - Perioperative hypotension:systolic blood pressure (SBP) < 80 mmHg or a decrease of 30% baseline value and was treated with reduction of anesthetics, fluid supplement, and a bolus dose of ephedrine (Mahuangsu™, Northeast Pharmaceutical Co., Ltd., Shenyang, CN) (10 mg, IV). If SBP was above 160 mmHg or increase > 30%, an increase of propofol or remifentanil infusion was given to deepen anesthesia.	- Monitoring: the same as fast-track group

-Antibiotic prophylaxis	- Yes,	- Yes

-Surgical management	-Laparoscopic/open surgery as randomization	-Laparoscopic/open surgery as randomization

- Warming	- Yes, body warming by thickening quilt as well as intravenous fluid warming	- No body and intravenous fluid warming

- Drains	- Minimal use and early removal of abdominal drains	-Regularly use and removal of abdominal drains

- Fluid infusion	- Totally ≤ 1,500 ml during operation	- No restriction

Postoperation		

- Pain management	-Patient-controlled continuous epidural analgesia with a 5 ml/h continuous infusion of 0.15% ropivacaine and a bolus dose of 2.5 ml (locktime 15 min) until 48 h after operation, paracetamol (Tylenol™, Johnson & Johnson Pharmaceutical Co., Ltd., Shanghai, CN) p.o. when needed	-Patient-controlled intravenous analgesia with a 4 ug/h continuous infusion of sufentanil and a bolus dose of 1.5 μg (locktime 15 min)
		
		-Bucinperazine (QiangtongdingTM, Northeast Pharmaceutical Co., Ltd., Shenyang, CN) or Morphine (Mafei™, Northeast Pharmaceutical Co., Ltd., Shenyang, CN) intramuscular injection when patient-controlled intravenous analgesia isn't enough for pain control

- Diet	- Chewing gum 1 piece tid p.o.	-No chewing gum
	
	- At least 10% Glucose 200 ml p.o. within 24 h after operation	- Fasting until flatus
	
	-Liquid diet and Enteral Nutritional Emulsion Supportan 200 ml or Fresubin Diabetes 300 ml (especially for patients with diabetes mellitus) p.o. the next day of operation	- Liquid diet after flatus
	
	- Diet rehabilitation as early as possible (dose increase of Enteral Nutritional Emulsion or when needed)	- Normal diet after defecation

- Intravenous fluid infusion	- Stop intravenous high energy fluid infusion after dosage of Enteral Nutritional Emulsion Supportan ≥ 600 ml or Enteral Nutritional Emulsion Fresubin Diabetes ≥ 500 ml	- Intravenous high energy fluid infusion on daily basis and continuing until adequate oral intake
	
	- No intravenous High-energy Nutrient Fluid after 72 h post-surgery	
	
	- Restricting and avoiding excessive intravenous fluid infusion, keeping body weight as pre-surgery	

- Energy	- Keep the total energy intake (both diet and intravenous fluid infusion) 25-30 kcal/kg/day	- Keep the total energy intake (both diet and intravenous fluid infusion) 25-30 kcal/kg/day

- Nasogastric tube and urethral catheter	-Remove nasogastric tube as soon as the end of operation	- Remove nasogastric tube after 1st flatus postoperation

	- Remove urethral catheter within 24-48 h after operation	-Remove urethral catheter when 1st time meet: patient have the feeling of automatic micturition and ≧200 ml after valving-on urethral catheter

- Ambulation	- Forced ambulation within 24 h post-surgery, no time restriction	- No ambulation scheme
	
	- Ambulation time ≥ 1 h per day, and increasing day by day	
	
	- Patients walking to weight themselves every day	

Adjuvant		

chemotherapy	- Xelox	- mFolfox6
	
	- repeat every 3 weeks for 8 cycles	- repeat every 2 weeks for 12 cycles
	
	- Regimen	- Regimen
	
	Oxaliplatin 130 mg/m2 day 1, Capecitabine (Xeloda™) 850-1,000 mg/m2 twice daily for 14 days -	Oxaliplatin (Eloxatin^TM^) 85 mg/m2 IV over 2 hours, day .1 Leucovorin (Tongao™) 400 mg/m2 IV over 2 hours, day 1. 5-FU (Jinyao™) 400 mg/m2 IV bolus on day 1, then 1,200 mg/m2/day × 2 days (total 2,400 mg/m2 over 46-48 hours) continuous infusion
	
	- No peripherally inserted central catheter (PICC)	- Peripherally inserted central catheter and care of PICC in outpatient clinic every week
	
	- Hospitalization no more than 24 h each cycle	- Hospitalization for 3 days each cycle

##### Adjuvant chemotherapy

In this study, adjuvant chemotherapy for III stage or high-risk II stage pathologically established colorectal cancer includes Xelox and mFolfox6, both of regimens which in accordance with NCCN Clinical Practice Guidelines in Oncology ™ Colon Cancer V.2.2009.

(a) mFolfox6 (repeat every 2 weeks for 12 cycles)

Oxaliplatin (Eloxatin™, Sanofi-aventis, Hangzhou, CN) 85 mg/m^2 ^IV over 2 hours, day 1

Leucovorin (Tongao™, Hengrui medicine Co., Ltd, Lian Yuan-gang, CN) 400 mg/m^2 ^IV over 2 hours, day 1

5-FU (Jinyao™, Tianjin KingYork Amino Acid Co., Ltd. Tianjin, CN) 400 mg/m^2 ^IV bolus on day 1, then 1,200 mg/m^2^/day × 2 days (total 2,400 mg/m^2 ^over 46-48 hours) continuous infusion

(b) Xelox, also known as CapeOX (repeat every 3 weeks for 8 cycles)

Oxaliplatin 130 mg/m^2 ^day 1, Capecitabine (Xeloda™, Roche, Shanghai, CN)

850-1,000 mg/m^2 ^twice daily for 14 days

##### Patient pathway

**Figure 1:**
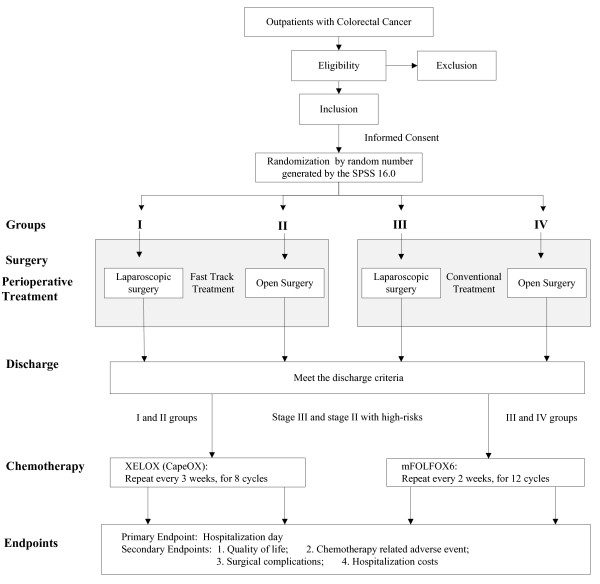
**The FTMDT trial flowchart**.

All the patients in the FTMDT trial will go through as depicted in the flowchart (Figure 1)

### Statistical analysis

#### Sample size calculation

Hospital stay is the primary endpoint. From the evidence in the researches [13,14,22-26], we estimate the hospital stay of laparoscopic and open surgery with Fast-track perioperative treatment is 6 and 8 days. Xelox chemotherapy has at most 1 hospitalization day for each cycle followed by taking Capecitabine orally at home, for a total of 8 cycles. Therefore, we calculate the hospital stay for groups I and II at 14 and 16 days. According to our previous research [27], the hospital stay of laparoscopic and open surgery with conventional perioperative treatment is 12 and 14 days. mFolfox6 chemotherapy requires at least 3 hospitalization days for each cycle, totally 12 cycles. So, we calculate the hospital stay of III and IV group is 48 and 50 days.

With the standard deviation of 6 days of mean hospitalization days, a total sample size of 218 would have a power of > 0.85 to detect a minimum reduction of 2 days in hospital stay among the 4 groups, using a 5% significance level. The III stage or high-risk II stage account for 64% of the total colorectal cancer patients [28], and these patients need the adjuvant chemotherapy according to the NCCN Clinical Practice Guidelines in Oncology-Colon Cancer Guideline 2009 (Version 2.2009) [29]. For this trial we will enroll 340 patients and have 85 patients in each group.

#### Economic evaluation

As one of the secondary outcomes, we would analyze the balance between the reduction of hospitalization days and the hospitalization cost, and evaluate whether or not FTMDT is a more economical colorectal disease treatment model.

#### Data collection and monitoring

All the data will be collected by assigned persons: the information of surgery and perioperative treatment will be collected during hospitalization, and the information of chemotherapy will be followed up.

A professional research associate develops regular contact between the study centers and monitors the information for each patient.

## Discussion

As we know, the surgery, perioperative treatment and chemotherapy for colorectal cancer stretches roughly to half a year at present, which means a long hospital stay for the patients and high medical resource costs.

As a result, two demand-oriented feasible methods have been brought into the colorectal cancer treatment, laparoscopic surgery and fast-track perioperative treatment.

For laparoscopic surgery, the COST Study Group trial has proved the advantages of laparoscopy for colon cancer, especially in faster perioperative recovery and shorter hospital stays, with the similar recurrence and overall survival rates to the open-colectomy group [1]. The UK MRC CLASICC Trial also demonstrated that laparoscopy-colectomy is as effective as open surgery in terms of oncological outcomes [30]. For Fast-track perioperative treatment, the LAFA Study Group and ERAS Group presented a systematic evidence-based consensus review, proving that Fast Track treatment appears to be safe and can shorten the hospital stay after elective colorectal surgery [9]. All the studies above provided the feasibility and safety of both laparoscopic surgery and fast-track perioperative treatment.

However, it is important to point out that, the enhanced recovery mentioned in current study about laparoscopy and Fast-track, is confined to the short-term postoperative recovery, which only lasts 1-2 weeks perioperatively. The previous study showed that quality of life benefits due to minimally invasive laparoscopic surgery were evident only in the immediate postoperative period [31]. As a result, the long-term benefit relies on the innovative treatment model covering not only surgery but also adjuvant chemotherapy. It's significant and interesting to investigate whether it will be possible to cover the enhanced recovery over the whole colorectal cancer treatment and how it works. Consequently, combining the long-term benefit chemotherapy treatment is taken into consideration.

The NO16968/XELOXA study discovered that the Xelox chemotherapy used as an adjuvant chemotherapy for colorectal cancer become possible now, as it has a manageable tolerability profile in adjuvant setting [17]. In each cycle of Xelox chemotherapy, there is only 1 day hospital stay for intravenous Oxaliplatin and the Capecitabine intake from 1st to14th day can be done in home. And in mFolfox6 chemotherapy, each cycle needs 3 days hospital stay in China. Consequently, it can be seen clearly that the superiority of Xelox for shorter hospital stay with the similar Overall Survival (OS) to mFolfox6.

On this consideration, before this trial, KF Ding has been conducting an open nonrandomized prospective study comparing laparoscopic surgery with Xelox chemotherapy and open surgery with mFolfox6 chemotherapy for resectable colorectal cancer, and the interim analysis has proved that the former provides faster postoperative recovery and potentially sustained better quality of life throughout treatment [32].

In the view of the separate superiority of the laparoscopy, Fast-track perioperative treatment and Xelox chemotherapy, and the possibility of the integration of these three essential treatments in colorectal cancer, the new notion "Fast Track Multi-Discipline Treatment" (FTMDT) for the integration of these three treatments is promoted in this FTMDT trial. And this FTMDT model is supposed to substantially reduce the length of hospital stays with equal safety and effectiveness, gain the better long-term recovery outcomes, and give patients a better quality of life.

The 2 × 2 balanced factorial design for this randomized prospective controlled FTMDT study tries to answer 2 corresponding questions. The I and II groups versus the III and IV groups is designed to answer the research question "Is Fast-track multidiscipline treatment model safe and effective, compared to conventional treatment?", while the I group versus II group aims to answer "Is laparoscopy an essential component of FTMDT-trial and how important role does it play?"

It's necessary to mention that, the second question about the necessity of laparoscopy use is closely related to the treatment cost. On one hand, the use of laparoscopy itself will increase the operation cost, compared to the open operation; on the other hand, the shorter hospital stay if the laparoscopy could bring, will conversely bring the reduction of the treatment cost. It is still unknown whether laparoscopic surgery has a beneficial effect on Fast-track perioperative treatment, although the most recent studies have proved that the laparoscopic surgery with Fast-track treatment also has shorter hospital stay and enhanced recovery [22-26]. However, research has found that there is no further benefit for the laparoscopy, compared with open surgery groups, and even with the two groups both using Fast-track perioperative treatment [33-35]. And some trials are still undergoing now to try to answer the question, like LAFA-trial which is now being conducted in Netherlands [36]. Consequently, it can be assumed that, if the laparoscopy did not play the essential role in enhanced recovery and reduced hospitalization, the use of laparoscopy in the Fast-track perioperative treatment could be considered again and the extra cost by laparoscopic appliance using may save.

The LAFA-trial which is now being conducted in the Netherlands has combined laparoscopy with fast track perioperative care, which will provide the merits of fast track perioperative care and laparoscopic colectomy [36]. Additionally, in this FTMDT trial the perioperative treatment of enhanced recovery for colorectal cancer treatment has the similar principle to the ERAS Group or LAFA Group [12,36], but involves the chemotherapy into study and also has a different definition for Fast track treatment. In this FTMDT trial, the notion of Fast Track treatment, is not only limited to the perioperative care but extended to the entirety of the colorectal cancer treatment, involving surgery, the perioperative treatment and the adjuvant chemotherapy. Correspondingly, the short-term superiority of the laparoscopic surgery and fast-track perioperative treatment, the long-term superiority of Xelox chemotherapy, may integrate together. Thereby, this FTMDT model covers the whole colorectal cancer treatment periods, makes it possible to achieve the whole process enhanced recovery and bring the economic benefits synonymously.

Concretely, at least 3 aspects of the advantage for the "Fast Track Multi-Discipline Treatment" Model are expected: 1. The whole process enhanced recovery: The FTMDT model itself covers the entire treatment processes for colorectal cancer. The patients who receive the laparoscopic surgery, fast-track perioperative treatment have enhanced short-term recovery, and can enter the chemotherapy earlier with good physical condition, and can also further earn the life quality benefits from the Xelox chemotherapy. 2. The economic benefits: The FTMDT model can substantially reduce the hospitalization stay because of enhanced recovery and convenient Xelox chemotherapy bring. The medical resources can be saved and the medical cost for colorectal cancer may decline as the result of decreased hospitalization stay. Moreover, Winterhalder reported Xelox is cost saving versus Folfox4, presenting the economic benefits of Xelox chemotherapy [37]. 3. The strengthened confidence of patients: The patients in the Fast Track Multi-Discipline Treatment arm earn the enhanced recovery, higher life quality, shorter hospital stay and the lighter economic burden, making patients the firmer faith against cancer and confidence for the further life.

In summary, this FTMDT trial gives insights into the potential merits and feasibility of the "Fast Track Multi-Discipline Treatment" model. A new guideline of whole process enhanced recovery treatment for colorectal cancer will be established according to the FTMDT model. This model will create another effective treatment pathway, bring economic benefits, and make the colorectal cancer treatment cost-saving and efficacy-gaining.

## Competing interests

The authors declare that they have no competing interests.

## Authors' contributions

KFD initiated and coordinated the study. KFD, JJZ and JL drafted the manuscript and took part in conducting the study and supervised the intervention. KFD, XJY, YMS and RC are responsible for the patient surgical treatment. MY and GC provide anesthesia treatment. All the authors have read and approved this final version of the manuscript.

## Authors' information

The principal investigator Prof. Ke-Feng Ding(MD/PhD) is director of the Surgical Oncology Department, 2nd Affiliated Hospital, School of Medicine, Zhejiang University, China. He is the PhD/MD supervisor of Zhejiang University. Dr. Ding currently serves as the member of Chinese Society of Oncology, the member and secretary of the Committee Colorectal Cancer, CACA, and the member of the Committee of Tumor Metastasis, CACA.

## Pre-publication history

The pre-publication history for this paper can be accessed here:

http://www.biomedcentral.com/1471-2407/11/494/prepub
